# Supplementary material for: Acute and chronic gregarisation are associated with distinct DNA methylation fingerprints in desert locusts
Source: Sci Rep. 2016 Oct 18;6:35608. doi: 10.1038/srep35608 (PMC5067648; doi:10.1038/srep35608)

Supplementary figures for:

Acute and chronic gregarisation are associated with distinct DNA methylation fingerprints in desert locusts

Eamonn B. Mallon[1], Harindra E. Amarasinghe[2], and Swidbert R. Ott[3*]

[1]Department of Genetics, University of Leicester, University Road, Leicester, LE1 7RH, United Kingdom.

[2]Academic Unit of Cancer Genomics, Faculty of Medicine, University of Southampton, Southampton, SO17 1BJ, United Kingdom.

[3]Department of Neuroscience, Psychology and Behaviour, University of Leicester, University Road, Leicester, LE1 7RH, United Kingdom.

[*]Corresponding author: S.R.Ott@cantab.net

Figures S1–S3 show the Principal Coordinate Analysis (PCoA) plots obtained with band scoring tolerance levels in the range of 11–25 bp.

Figure S1. Principal Coordinate Analysis (PCoA) of epigenetic differentiation between uncrowded solitary-reared locusts (Solitarious), long-term gregarious locusts (Gregarious) and solitary-reared locusts crowded for 24 h (Behaviourally gregarious), as identified by MS-AFLP for tolerances of 11–16 bp (a–f). The first two coordinates (C1, C2) are shown with the percentage of variance explained by them. Group labels show the centroid for each group, points correspond to individual MS-AFLP samples, ellipses represent their average dispersion around the group centroids.

(a) Tolerance = 11 bp

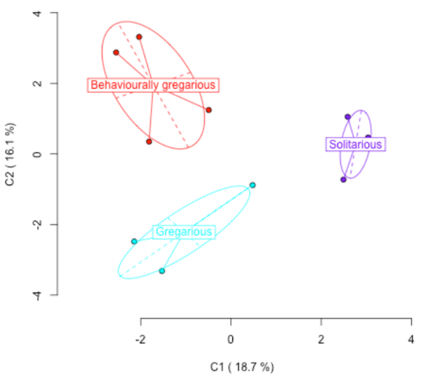

(b) Tolerance = 12 bp

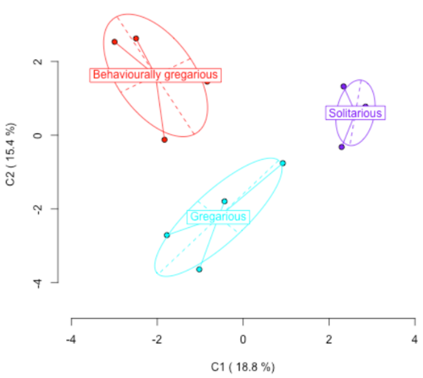

(c) Tolerance = 13 bp

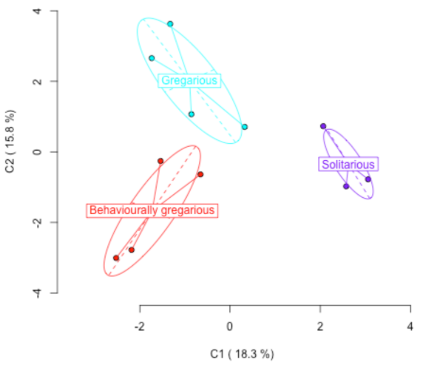

(d) Tolerance = 14 bp

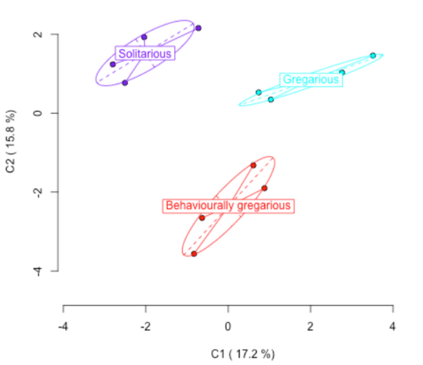

(e) Tolerance = 15 bp

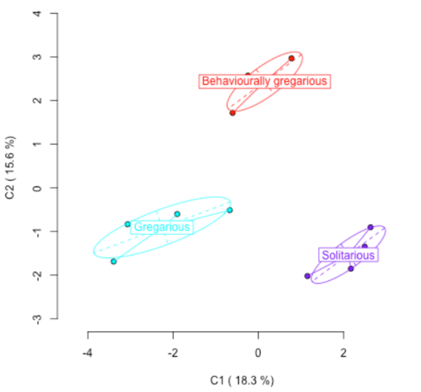

(f) Tolerance = 16 bp

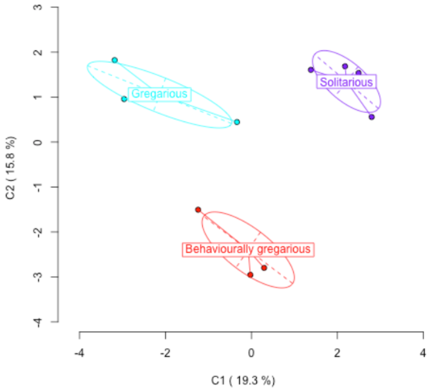

Figure S2. Principal Coordinate Analysis (PCoA) of epigenetic differentiation between uncrowded solitary-reared locusts (Solitarious), long-term gregarious locusts (Gregarious) and solitary-reared locusts crowded for 24 h (Behaviourally gregarious), as identified by MS-AFLP for tolerances of 17–22 bp (a–f). The first two coordinates (C1, C2) are shown with the percentage of variance explained by them. Group labels show the centroid for each group, points correspond to individual MS-AFLP samples, ellipses represent their average dispersion around the group centroids.

(a) Tolerance = 17 bp

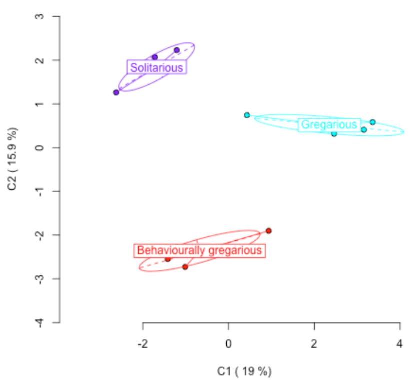

(b) Tolerance = 18 bp

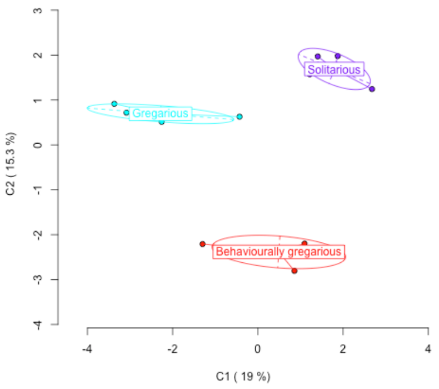

(c) Tolerance = 19 bp

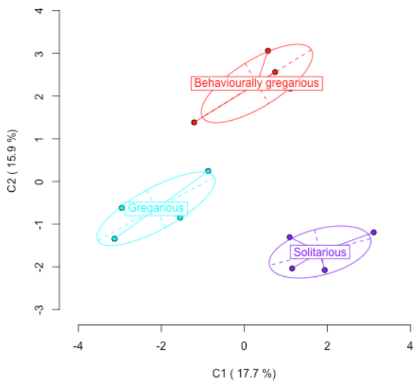

(d) Tolerance = 20 bp

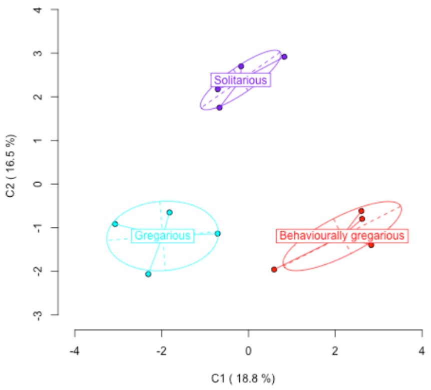

(e) Tolerance = 21 bp

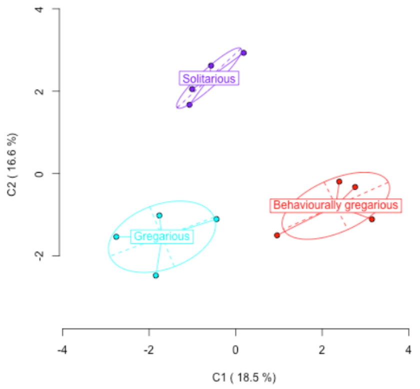

(f) Tolerance = 22 bp

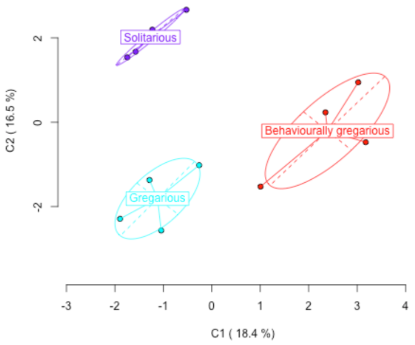

Figure S3. Principal Coordinate Analysis (PCoA) of epigenetic differentiation between uncrowded solitary-reared locusts (Solitarious), long-term gregarious locusts (Gregarious) and solitary-reared locusts crowded for 24 h (Behaviourally gregarious), as identified by MS-AFLP for tolerances of 23–25 bp (a–c). The first two coordinates (C1, C2) are shown with the percentage of variance explained by them. Group labels show the centroid for each group, points correspond to individual MS-AFLP samples, ellipses represent their average dispersion around the group centroids.

(a) Tolerance = 23 bp

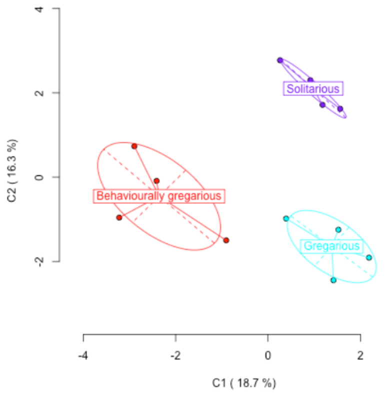

(b) Tolerance = 24 bp

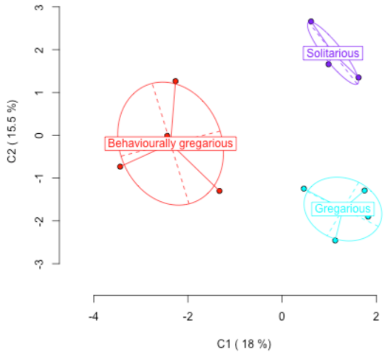

(c) Tolerance = 25 bp

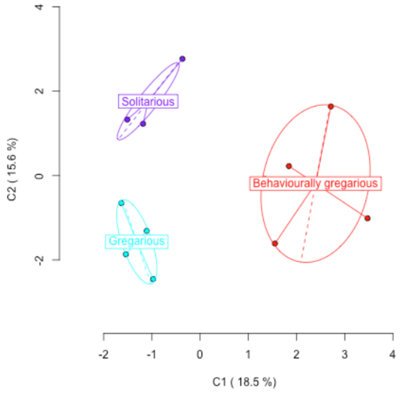

Supplement: Supplementary Information [file srep35608-s1.pdf]
